# A case of visceral leishmaniasis found by left oblique hernia: A case report

**DOI:** 10.3892/etm.2020.9477

**Published:** 2020-11-17

**Authors:** Guoqiang Zhang, Jihua Zhong, Ting Wang, Lu Zhong

Exp Ther Med 19:2697–2701, 2020; DOI: 10.3892/etm.2020.8487

After the publication of the above paper, the authors have realized that the address presented for the authors, and the email address for the corresponding author, were both published containing errors. First, the address affiliation should have been presented as “Department of Hematology, Renji Hospital, **School of Medicine**, Shanghai Jiaotong University, Shanghai **200127**, P.R. China” (instead of as “Department of Hematology, Renji Hospital, Medical College, Shanghai Jiaotong University, Shanghai 200001, P.R. China”). Secondly, the email of the corresponding author should have been presented as “j**h**zhong28@163.com” (instead of as “jizhong28@163.com”). Therefore, the authors’ names and affiliations in this paper should have been presented as follows (changes are highlighted in bold):

GUOQIANG ZHANG, JIHUA ZHONG, TING WANG and LU ZHONG

Department of Hematology, Renji Hospital, **School of Medicine**, Shanghai Jiaotong University, Shanghai **200127**, P.R. China

*Correspondence to*: Professor Jihua Zhong, Department of Hematology, Renji Hospital, **School of Medicine**, Shanghai Jiaotong University, 160 Pujian Road, Pudong New Area, Shanghai **200127**, P.R. China

E-mail: jhzhong28@163.com

The authors regret that these errors with the author affiliations and the corresponding author’s email address were not noticed prior to the publication of their paper, and apologize for any inconvenience caused.

